# Evaluation of the Tunnelling Technique for Upper Labial Frenectomy Using ER:YAG Laser

**DOI:** 10.4317/jced.63544

**Published:** 2026-03-30

**Authors:** Luis Monteiro, Inês Álvaro, Sara Ferreira, Leonor Delgado, Teresa Vale, José Júlio Pacheco, Filomena Salazar

**Affiliations:** 1 UNIPRO, Oral Pathology and Rehabilitation Research Unit, University Institute of Health Sciences (IUCS-CESPU), Gandra 4585-116, Portugal; 2 Medicine and Oral Surgery Department, University Institute of Health Sciences (IUCS), CESPU, Gandra 4585-116, Portugal; 3 GIPOC-Comparative Oral Pathology Research Group, University Institute of Health Sciences-Advanced Polytechnic and University Cooperative (IUCS-CESPU), 4585-116 Gandra, Portugal

## Abstract

**Background:**

An abnormal upper labial frenum (ULF) can represent a potentially harmful gingival insertion located between the central incisors. Lasers, particularly the Er:YAG laser, have been described for ULF frenectomy and offer several promising advantages. The aim of this study was to evaluate the usefulness of Er:YAG laser frenectomy using the "tunnelization technique" for the elimination of an abnormal upper labial frenum.

**Material and Methods:**

This cohort study evaluated ULF frenectomy using the tunnelling technique with an Er:YAG laser in a cohort of 20 patients treated between January 2011 and December 2024. The ULFs were morphologically and clinically characterized before and after the procedure.

**Results:**

No intraoperative complications were observed during the frenectomy, apart from moderate intraoperative bleeding in 12 patients (60%), with no sutures required in any case. Postoperatively, analgesic medication was required by only 2 patients (10%) for mild pain. At the 3-month follow-up, a significant reduction was observed in both frenulum size (P&lt;0.001) and interincisal diastema (P=0.008). All incisive papillae were preserved without any defects.

**Conclusions:**

Er:YAG laser frenectomy using the tunneling technique proved to be a suitable option, resulting in the immediate preservation of the incisive papilla and a reduction in ULF size and position, without significant associated complications.

## Introduction

The upper labial frenulum (ULF) consists of an anatomical structure composed of fibrous, muscular, or fibromuscular tissue, extending from the inner surface of the upper lip to its insertion at the midline of the interincisal gingival tissue at the mucogingival junction (1 - 4). The function of the frenulum is to limit and stabilize the lip movements, preventing excessive exposure of the gingival mucosa (2). The frenulum can present an abnormal insertion between the central incisors, with a progressively coronal position and can be characterized as abnormal when its structure is hypertrophic, broad, fibrotic-rigid, fan-shaped, or bifid-ended (5). It may restrict lip mobility, limiting mastication and phonetics, limit facial mimicry and aesthetics, as well as causing clinical, prosthetic, periodontal, and orthodontic problems, increasing the risk of gingival recession, and gingivitis development, impairing proper tooth brushing (1 - 4). Moreover, abnormalities in frenulum size and location can lead to the development and persistence of an interincisal diastema (6). Papillary and penetrating papilla insertions frequently cause "pull syndrome," characterized by ischemia of the palatal papilla and mesial gingival margins of the upper central incisors upon traction of the upper lip (1 , 7). Depending on the insertion of the frenulum on mucosa, it can be classified anatomically by several types. The Mirko et al.'s classification (8) presents a frenulum classification system divided into four categories based on its insertion: type I- mucosa (including mucogingival junction); type II- gingival insertion; type III- interdental papilla insertion; and type IV- transpapillary insertion. According to Monti et al.'s classification (3), frenula are classified as elongated with parallel margins (type I), triangular with an apical base (type II), and triangular with a coronal base (type III). Diagnosis is based on multiple clinical and imagiologic aspects. For example, a blanching test could be used, applying tension to the frenulum to observe movement of the papilla tip and detect whitening due to ischemia. Periapical X-rays can show an inverted V-shape defect specially on cases with penetrating papilla insertions (6 , 9 , 10). Removal of abnormal frenulum is often indicated in cases of persistent midline diastema after eruption of permanent canines, or by orthodontic or periodontal problems (9 , 11). Treatment involves frenectomy, aiming to eliminate excess interdental tissue, and can be performed using conventional scalpel technique, electrosurgery, or lasers (1 , 2 , 7). Advantages of laser surgery include a bloodless surgical field, no need for suturing, reduced pain and edema, disinfection of the operative field, precision in tissue excision with less damage to adjacent tissues, and enhanced hemostasis (1 - 3 , 5). The most commonly used lasers are diode lasers (600-980 nm), carbon dioxide (CO2) lasers, (10,600 nm), neodymium-doped yttrium aluminum garnet (Nd:YAG) lasers (1064 nm), and erbium-doped yttrium aluminum garnet (Er:YAG) lasers (2940 nm), as well as Er,Cr:YSGG lasers (erbium-chromium-yttrium scandium-gallium garnet) (1 , 7 , 12 , 13). The Er:YAG laser has the capability to cut mucosal tissue as well as periosteum due to its affinity for water and hydroxyapatite, thus enabling removal of deep periosteal insertions (5 , 14). In the view of this, this laser wavelength could be a good option for the ablation not only of the mucosal visible frenulum but also for the internal intraseptal fibers below incisal papillae often located in interincisive suture. The primary aim of this study was to evaluate the efficacy of Er:YAG laser frenectomy using the tunneling technique for the elimination of the components of an abnormal upper labial frenum (ULF). Secondary aims included assessing the reinsertion of the ULF following the procedure, evaluating the associated clinical and potential complications before and after surgery, and monitoring for early frenulum recurrence.

## Materials and Methods

**Figure 1:**
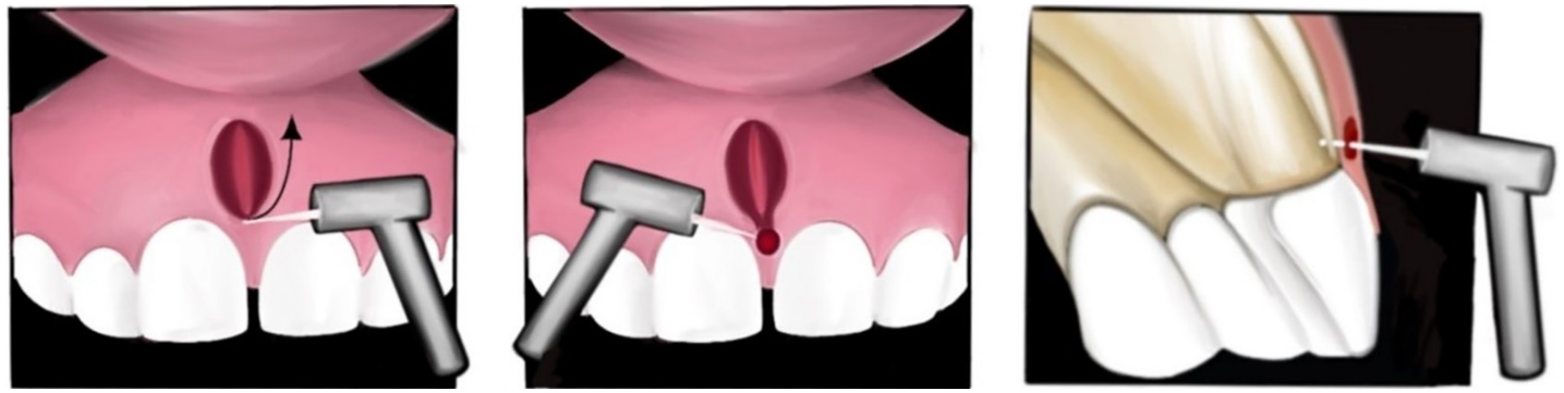
Illustration of the frenectomy using the tunnelling technique performed with Er:YAG (2940nm) laser.

1. Type of study, Research Details and Classification This was a retrospective observational cohort study, conducted at the University Clinics of Instituto Universitário de Ciências da Saúde do Norte (IUCS - CESPU). It included patients who underwent Er:YAG laser frenectomy using the Tunnelling technique between January 2011 and December 2024. The study received approval from the Ethics Committee of IUCS - CESPU (06/CE-IUCS/2025), and all procedures adhered to the ethical guidelines for clinical research involving human subjects as outlined in the Declaration of Helsinki. All patient data and information were anonymously recorded, and every participant or their legal representative signed an informed consent form. For each patient, medical history was taken, along with orthopantomography and a periapical radiograph of the upper inter-incisal space. Included patients were referred from orthodontics, pediatric dentistry, and oral pathology specialty consultations. These patients had an indication for ULF frenectomy due to an abnormal ULF or for orthodontic reasons. Inclusion criteria also required a Type III (interdental papilla insertion) or Type IV (transpapillary insertion) frenum according to Mirko et al. (8) classification. Patients were excluded if there was no reason for frenectomy, an ASA classification of IV or higher, an inter-incisal diastema due to causes other than improper upper labial frenum insertion, current use of anti-inflammatory drugs, antibiotics, or analgesics at the time of surgery, or a history of oral surgery, systemic diseases, or any oral lesion within 30 days prior to the surgery date. 2. Laser settings A 2,940 nm Er:YAG laser (LightWalker, Fotona, Slovenia) with an R14 handpiece featuring an 8/1.3 mm cylindrical sapphire tip was used. The laser settings were: pulse energy of 0.120 J, a repetition rate of 20 Hz (resulting in an output power of 2.4 W), and a very long pulse (VLP) mode. Initially, water and air spray were used, transitioning to no spray at the end for hemostasis control. All laser safety measures were followed, with all professionals and the patient wearing protective glasses covering the 2,940 nm wavelength. 3. Surgical Procedure and description of the Tunnelling technique Patients were initially anesthetized using bilateral vestibular infiltration technique with XILONIBSA 2% with epinephrine 1:80,000 (INIBSA®). After anaesthesia, the upper lip was lifted to stretch the frenum (Fig. 1).

1

An initial incision was made in the vertical plane along the frenum's axis, followed by a V-shaped design that traced the frenum's insertion and the mucogingival junction, creating a small rhomboidal surgical wound. The visible fibrous tissue of the frenum was ablated. For frenula with insertion below the incisive papilla, a superficial tissue ablation was performed, avoiding any cutting or decussation of the papilla. The second step involved (using a short pulse mode with spray settings) the remotion of intraseptal fibrous tissue from the superior aspect of the interincisal bone crest. This was achieved by local laser mucosal perforation, approximately 1-mm in depth, starting superior to the papilla and extending to the bone crest, and then circling the bone papilla from anterior to posterior to ensure the desinsertion of collagen fibers. Although no perforation of the palatal mucosa is expected, a protective plastic "mirror-like" instrument was used to prevent any laser reflection or scattering during the procedure. In a third step, hemostasis was verified. If spontaneous hemostasis was not achieved, a suture was performed. All treatments were performed by the same operator (LM) with clinical and academic experience in laser field. 4. Data Collection and Evaluation Instruments Before the start of the surgery, all frenula were classified according to Mirko et al. (8) and Monti et al. (3) classification. The measurement of the distance from the tip of the frenulum insertion to the mucogingival line and of diastema width was recorded using a two-point caliper. Ischemia of the incisive papilla upon traction was also evaluated. Subsequently, orthopantomography and a periapical X-ray of the diastema area were performed. During surgery, the duration of the procedure was recorded in minutes from the moment of incision until final hemostasis. Intraoperative bleeding was recorded in all patients as: 0 - No bleeding, 1- mild bleeding, 2- moderate bleeding, and 3 - severe bleeding (16). Intraoperative pain was assessed using the Visual Analog Scale (VAS) (17) used to classify the pain felt by each patient and represented by a numerical pain scale ranging between two intervals, "no pain" (0) and "worst imaginable pain" (10). At the end of the surgery, immediately, it was evaluated whether suturing was necessary and reported any potential complications of surgery. Postoperative follow-up visits were carried out on the 7th day (1st follow-up visit), 21st day (2nd follow-up visit), 30th day (3rd follow-up visit) and 90th day (4th months visit). At the 3rd and 4th visit, all postoperative frenula were again classified according to their insertion (Mirko et al. classification) (8) and according to the Monti classification (3). During last visit, the measurement of the distance from the frenulum insertion to the mucogingival line, and the postoperative diastema width was made to evaluate the potential diastema closure. For the evaluation of wound healing, the day of the follow-up visits when most patients presented complete healing was recorded. Postoperative pain was recorded up to the during follow-up visits, evaluated using the same methodology as for intraoperative pain. All patients were instructed on postoperative care and good oral hygiene. Patients were advised to take analgesics if postoperative pain justified it. Patients were provided with information regarding the visual appearance of the surgical wound, as it could present a white exudate during the secondary intention healing process. 5. Statistic Analysis Statistical analysis was performed using the SPSS® program, version 30.0. A power analysis was performed to determine the adequacy of the study group using G Power software (v. 3.1.9.7). The sample size was determined based on the expected change in the distance from the frenulum insertion to the mucogingival line. Considering a significance level of 5% ( = 0.05) and a statistical power of 80% (1 - = 0.80), to detect a large effect size (Cohen's dz = 0.8) in a paired-samples t-test, a minimum of 15 patients would be required. Data were analysed using descriptive statistical techniques and comparative analysis between groups. The Mann-Whitney test was used in the comparisons between categoric groups and Kruskal-Wallis test for continuous variables groups. For the intragroup comparisons we used the Friedman test. The level of significance used for the statistical tests was 5%.

## Results

The study sample comprised a cohort of 20 patients with a mean age of 12.5 ± 5.7 years (range: 7 to 25 years). Of these, 15 (75%) were female and 5 (25%) were male (Table 1).

Table 1

**Figure 2:**
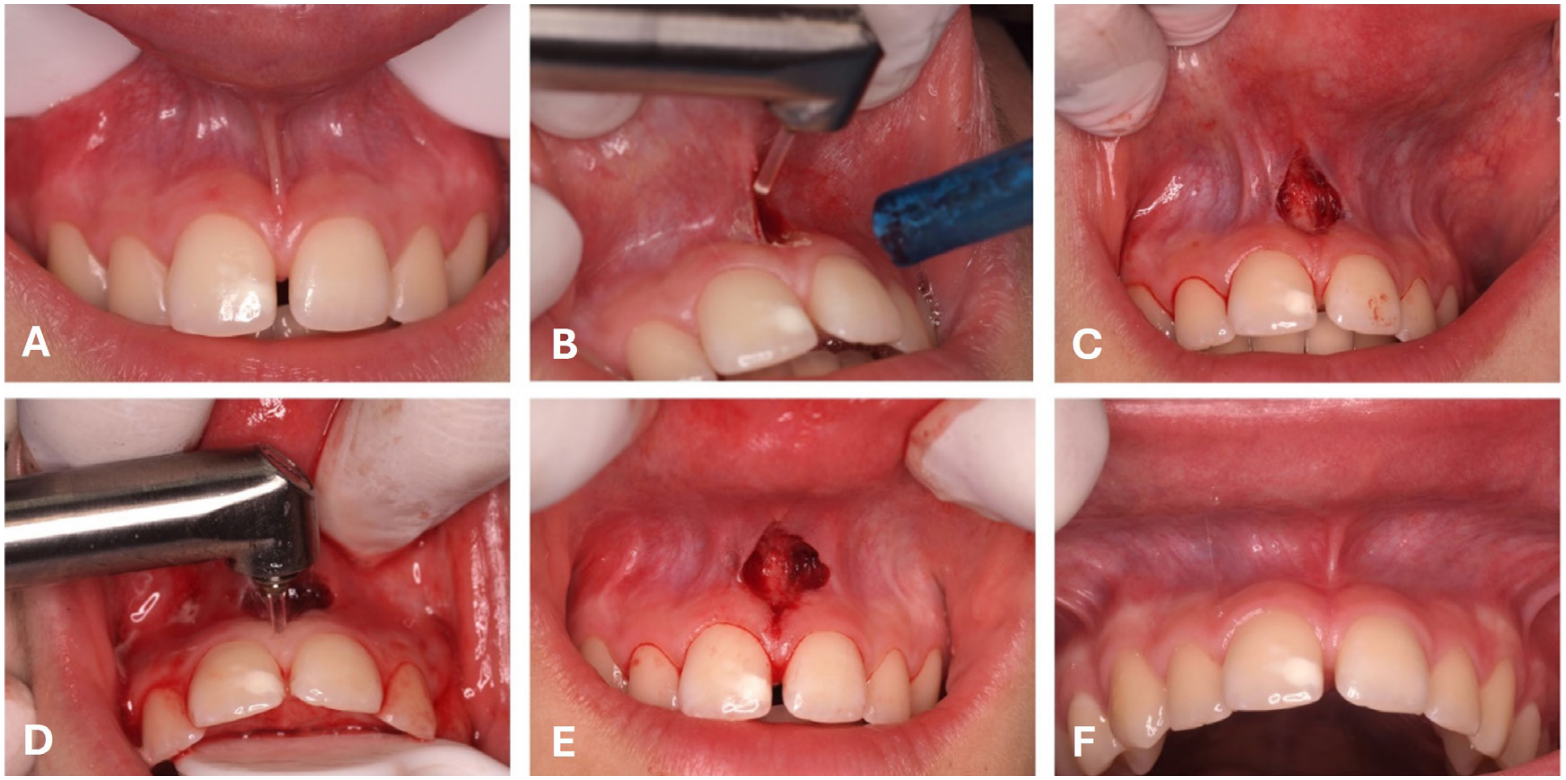
Images of a case of a female 18 years-old patient with a Type IV labial frenula, with diastema (A). First step of Frenectomy using a Er:YAG laser performing a vertical plane along the frenum’s axis, followed by a V-shaped design (B), resulting in a small rhomboidal surgical wound (C). The second step involved local laser mucosal perforation, extending to the bone crest, and then circling the bone papilla to ensure the desinsertion of collagen fibers (D). In a third step, hemostasis was verified with no need for a suture (D). After 21 days of follow-up there was were no complications and the position of the frenelum was before the mucogingival line and classified as a type I of Mirko et al.’s classification.

All patients had at least one permanent maxillary central incisor erupted, while 9 (45%) presented with erupted canines. Labial frenulum morphology, classified according to Mirko et al. (8), was predominantly Type III, observed in 11 (55%) patients (Table 1). Based on the Monti et al. classification (3), Type II frenulum was the most common, found in 13 patients (65%). Ischemia of the incisive papilla upon traction occurred in 7 patients (35%); of these, 6 (85.7%) had a Type IV frenulum according to Mirko et al. (8) (P=0.017). The mean distance from the frenulum insertion to the mucogingival line (MGL) was 4.23 ± 1.59 mm. In patients presenting with a diastema, the mean diastema width was 1.64 ± 1.09 mm. The mean surgical time was 9.15 ± 2.96 minutes (range: 4 to 14 minutes). During surgery, only one (5%) patient reported mild pain/discomfort (VAS of 1). No intraoperative complications were observed. Intraoperative bleeding was classified as moderate in 12 patients (60%), mild in 7 patients (35%), and severe in one patient (5%). Nevertheless, at the end of surgery, hemostasis was achieved in all patients without requiring additional measures (Fig. 2).

2

**Figure 3:**
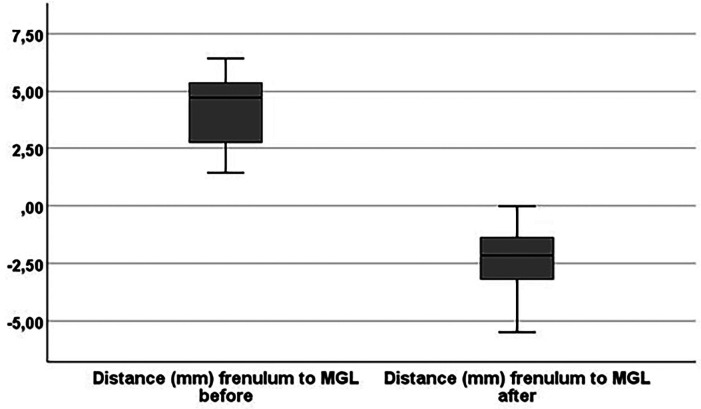
Boxplot of distances from the insertion of frenulum to mucogingival line (MGL) before and three months after the frenectomy (Test Friedman P &lt;0.001).

Suturing was not required in any patient. Postoperatively, only 2 (10%) patients reported pain: one (5%) experienced mild pain two days after surgery (VAS of 1 out 10), and the other (6.7%) reported pain (VAS of 3 out 10) three days after surgery. Analgesic medication (paracetamol) was required only by these 2 patients (10%). At the 7-day follow-up, no pain, bleeding, or discomfort was reported. By 21 days, all surgical wounds were completely healed in all patients. At the 3-month follow-up, the distance from the frenulum insertion to the mucogingival junction (MGJ) was compared to the preoperative measurement. A significant reduction in this distance was observed (P &lt; 0.001) (Table 2) (Fig. 3).

Table 2

3

**Figure 4:**
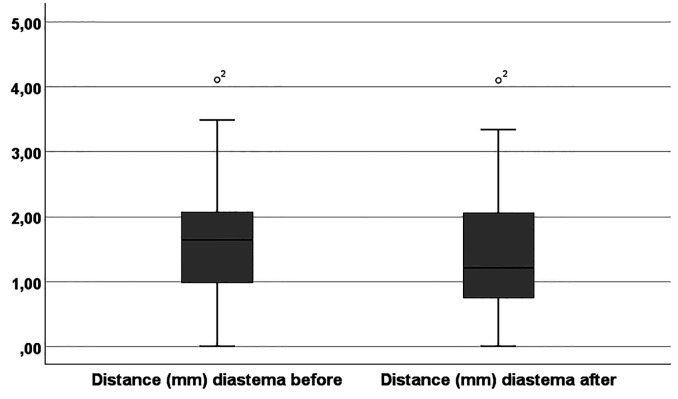
Boxplot of interincisive diastema distances before and three months after the frenectomy (Test Friedman P=0.008).

Postoperatively, in all patients, the frenulum insertion was located on the vestibular oral mucosa or at the MGJ. According to the Mirko et al., (8) classification, almost the frenula (19; 95%) were now classified as Type I (positioned superior to the MGJ). A small but significant reduction in diastema width was also observed (P = 0.008) (Fig. 4).

4

No frenulum recurrence was observed during the follow-up period. All incisive papillae were preserved without any defects.

## Discussion

The aim of this work was to report the utility of the frenectomy using the tunnelling technique performed with Er:YAG (2940nm) laser focusing the existence of potential complications associated and early outcome of this method. In this cohort of patients, this technique efficiently removed anomalous frenulum, in a short period of operatory time, without significant complications such as intraoperative and postoperative pain, or early recurrence and with an excellent tissue healing. The operative time in the present study, from the initial incision to end of procedure was 8.53 ± 3.04 minutes (ranging from 4 to 14 minutes). Samardi et al., (4) found that surgery time in their laser group close to 7 minutes shorter than with the conventional scalpel technique. Others have reported even shorter surgical times specially when using lasers with more photothermal effects (3). While our operative time was slightly longer, this could be attributed to the inclusion of an additional step involving the elimination of intraseptal fibers via tunnelization, a step that is often omitted in ellectroscalpel or laser frenectomies and that is important for a good outcome in a Type III or Type IV Mirko et al., (8) frenectomies (18). Report of Pain was almost absent, observed in only one and two patients and two patients, intraoperative postoperative, respectively, as observed by other authors (4 , 17). Some studies have documented higher levels of postoperative pain and discomfort frenectomies performed with scalpel compared to laser cases (1). This could be related with properties of lasers specially Er:YAG lasers (16) and related with a lower need of the use of analgesics after surgery as reported by Devishree et al., (9) and El Mobadder et al., (19). In the line with the results of present study and confirming one of the main advantages of laser surgery, there was a reduction of the use of analgesics and reduction of postoperative pain. Nevertheless, ER:YAG laser, has not the hemostasis potential of higher thermal lasers such as CO2 and Nd:YAG lasers explaining the presence of moderate bleeding during the procedure in some patients of our sample. This has been reported in other studies. Suter et al., (20) observed that intra-operative bleeding was higher in patients undergoing ER:YAG surgery than in those undergoing CO2 laser surgery. Pie-Sanchez et al., (3) observed a bloodless field using CO2 lasers compared to the ER,CR:YSGG laser group, which presented some bleeding. However, no suturing was necessary in any patient of our sample, as observed by other authors (17). As observed by others, surgical wounds exhibited complete healing by day 21 (3 , 5 , 18 , 21). Er:YAG laser has been related with an increasing collagen synthesis and accelerating healing of the injured periodontal ligament promoting an excellent healing of the tissues (22). On follow-up evaluation, the insertion of the frenum became located superior to the mucogingival junction (MGJ) in almost cases. A significant reduction in the interincisal diastema width was also observed (P=0.008) at 3 months. This was observed also in other studies in a follow-up time of 6 months (23). However, Sarmadi et al., (4) at the 3-month follow-up, reported that the interincisal diastema closed more, in mm, in the scalpel group than in the ER:YAG laser group, although the result was not highly significant. This could be related with type of technique used in performing the frenectomy (complete vs partial) as described before. However, as stated by Suter et al., (10) frenectomy using lasers has been proposed as a method to assist in closing the upper interincisal diastema, and combining frenectomy with orthodontic treatment could be more effective than isolated upper labial frenectomy. The use of a Tunnelling technique, performed with the ER:YAG laser, could be an less-invasive option to achieved complete ablation of the deep periosteal fibers (circular, transeptal, and interseptal fibers) inserted at the deepest bone level in this region, as well as removal of fibrous tissue from the insertion site at the mucogingival junction to the insertion of the frenum in the palatal region. This is possible using a Er:YAG laser with affinity by water and hydroxyapatite (5 , 12) allowing the efficient cut of gingival mucosae, periossium and even the fibers inserted at a deeper bone level both interincisally and palatally without thermal damage when comparing with other instruments (2 , 12). It is well described that the elimination of circular and transeptal fibers inserted at a deeper bone level, is an important step of an efficient frenectomy (18). An important aspect of our Tunnelling technique is the preservation of the interincisal papilla, without decapitation, as the Tunnelling perforation avoids reaching its structural limits and thereby prevents anatomical damage allowing the remotion of interseptal fibers using a sapphire tip. Additionally, the less-invasive and more conservative frenectomy of soft tissue can lead to less tissue scaring when compared with other techniques such as V-shaped/Archer incision method or Z-plasty technique (7 , 24). With the removal of the deep periosteal fibers, the likelihood of recurrence of the abnormal frenum is reduced, thereby allowing for diastema closure - as observed in the present study, where no recurrence was recorded at 3-month follow-up visits and a significant reduction in interincisal diastema was achieved. It also improves aesthetics, as the incision is minimal with a good prognosis due to the high reepithelialization capacity and no decapitation of the interincisal papilla. We acknowledge some limitations this study. Firstly, the intrinsic limitations of retrospective studies, including the potential for subjective evaluation of some variables and the lack of information of some variables. The sample size could have been larger to provide greater scientific robustness. Another limitation of this study is the short follow-up period for some patients, which prevents us from drawing conclusions about long-term outcomes. The lack of control group also impairs some discussions that we could make regarding the best instruments to perform upper lip frenectomy. Nevertheless, because the laser procedures were performed within a specialized Laser Unit, several variables were systematically and homogeneously reported. This article described for the first time the frenectomy using the Tunnelling technique performed with Er:YAG (2940nm) laser with removal of deep periosteal fibers, a potential problem when using many of lasers on frenectomies techniques. However, the authors plan to address these limitations in the future by conducting a prospective clinical trial with randomized groups exploring different treatment options for comparison and with a long follow-up evaluation to confirm the present results.

## Conclusions

According to the results of the present study, the Tunnelling technique performed with the ER:YAG laser is a useful method for performing upper labial frenectomy eliminating the anomalous/pathological frenula, in a reduced period of time and without significant complications. All cases showed a reinsertion on vestibular mucosa and with preservation of the interincisal papilla, within 21 days of follow-up. No recurrence and significant reduction of interincisal diastema was observed in 3 months of follow-up. Further studies with longer follow-up time and with randomized controlled arm of different instruments should be developed to confirm the results of the present study to confirm our results.

## Figures and Tables

**Table 1:** Characteristics of included patients in the study.

Variable	Category	Statistic Reported	Female(n=15)	Male(n=5)	Total(n=20)	P-value
Age (year-old)		Mean	12.20	13.40	12.50	0.695
		Standard Deviation	5.78	6.02	5.70	
Mirko's Classification	III	Count	8	3	11	0.604
		% within Mirko's Classification	72.7%	27.3%	100.0%	
		% within Gender	53.3%	60.0%	55%	
	IV	Count	7	2	9	
		% within Mirko's Classification	77.8%	22.2%	100.0%	
		% within Gender	46.7%	40.0%	45%	
	Total	Count	15	5	20	
		% within Mirko's Classification	75%	25%	100.0%	
		% within Gender	100.0%	100.0%	100.0%	
Monti's Classification	I	Count	3	3	6	0.481
		% within Monti's Classification	50.0%	50.0%	100.0%	
		% within Gender	20.0%	60.0%	30%	
	II	Count	12	1	13	
		% within Monti's Classification	92.3%	7.7%	100.0%	
		% within Gender	80%	20.0%	65%	
	III	Count	0	1	1	
		% within Monti's Classification	0.0%	100.0%	100.0%	
		% within Gender	0.0%	20.0%	5%	
	Total	Count	15	5	20	
		% within Monti's Classification	75%	25%	100.0%	
		% within Gender	100.0%	100.0%	100.0%	
Distance (mm) frenulum to MGL (before)		Mean	4.46	3.51	4.23	0.260
		Standard Deviation	1.44	1.97	1.59	
Distance (mm) diastema (before)		Mean	1.46	2.16	1.64	0.228
		Standard Deviation	0.98	1.37	1.09	

1

**Table 2:** Distances related with frenulum before and after frenectomy. MGL, mucogingival line.

Variable	N	Mean	Standard Deviation	Minimum	Maximum	P-value
Distance(mm) frenulum to MGL (before)	20	4.230	1.5940	1.44	6.43	< 0.001
Distance(mm) frenulum to MGL (after)	20	-2.2994	1.31266	0	-5.48	
Distance(mm) diastema (before)	20	1.6410	1.09635	0	4.11	0.008
Distance(mm) diastema (after)	20	1.439	1.1423	0	4.10	

2
